# Preoperative CT versus diffusion weighted magnetic resonance imaging of the liver in patients with rectal cancer; a prospective randomized trial

**DOI:** 10.7717/peerj.1532

**Published:** 2016-01-14

**Authors:** Michael P. Achiam, Vibeke B. Løgager, Bjørn Skjoldbye, Jakob M. Møller, Torben Lorenzen, Vera L. Rasmussen, Henrik S. Thomsen, Talie H. Mollerup, Cecilie Okholm, Jacob Rosenberg

**Affiliations:** 1 Department of Surgical Gastroenterology, Abdominal Centret, Rigshospitalet, University of Copenhagen, Copenhagen, Denmark; 4 Department of Radiology, Herlev Hospital, University of Copenhagen, Herlev, Denmark; 5 Department of Surgical Gastroenterology, Herlev Hospital, University of Copenhagen, Herlev, Denmark

**Keywords:** Contrast enhanced ultrasound, Magnetic resonance imaging, Metastasis rectal cancer, Diffusion weighted magnetic resonance, Computed tomography

## Abstract

**Introduction**. Colorectal cancer is one of the most frequent cancers in the world and liver metastases are seen in up to 19% of patients with colorectal cancers. Detection of liver metastases is not only vital for sufficient treatment and survival, but also for a better estimation of prognosis. The aim of this study was to evaluate the feasibility of diffusion weighted MRI of the liver as part of a combined MR evaluation of patients with rectal cancers and compare it with the standard preoperative evaluation of the liver with CT.

**Methods**. Consecutive patients diagnosed with rectal cancers were asked to participate in the study. Preoperative CT and diffusion weighted MR (DWMR) were compared to contrast enhanced laparoscopic ultrasound (CELUS).

**Results**. A total of 35 patients were included, 15 patients in Group-1 having the standard CT evaluation of the liver and 20 patients in Group-2 having the standard CT evaluation of the liver and DWMR of the liver. Compared with CELUS, the per-patient sensitivity/specificity was 50/100% for CT, and for DWMR: 100/94% and 100/100% for Reader 1 and 2, respectively. The per-lesion sensitivity of CT and DWMR were 17% and 89%, respectively compared with CELUS. Furthermore, one patient had non-resectable metastases after DWMR despite being diagnosed with resectable metastases after CT. Another patient was diagnosed with multiple liver metastases during CELUS, despite a negative CT-scan.

**Discussion**. DWMR is feasible for preoperative evaluation of liver metastases. The current standard preoperative evaluation with CT-scan results in disadvantages like missed metastases and futile operations. We recommend that patients with rectal cancer, who are scheduled for MR of the rectum, should have a DWMR of the liver performed at the same time.

## Introduction

Colorectal cancer is one of the most frequent cancers in the world with approximately 1.4 million new cases and 700.000 estimated deaths in the 2012 worldwide (International Agency for Research on Cancer, 2012). Liver metastases are seen in up to 19% of patients with colorectal cancers and other 14–20% metachronous liver metastases are seen during the subsequent five years for patients without initial synchronous metastases (Ghiringhelli et al., 2014; Landreau et al., 2015; Manfredi et al., 2006; Leporrier et al., 2006).

Detection of liver metastases is not only vital for sufficient treatment and survival, but also for a better estimation of prognosis. Currently, the best treatment and chance of survival for patients with liver metastases, both synchronous and metachronous, is either surgery or radiofrequency ablation (RFA). The five year overall survival with synchronous liver metastases without treatment at this time is approximately 3.3% (Manfredi et al., 2006), compared with a 5-year overall survival of 46% for patients with resected synchronous liver metastases (Harmon et al., 1999). Although the rate of synchronous liver metastases have been stable over the last decades, a decrease in metachronous metastases has been seen primarily due to better neo-adjuvant treatment (Leporrier et al., 2006). This underlines the importance of preoperative evaluation in patients with colorectal cancer in order to offer the best treatment: resection, neo-adjuvant chemotherapy or palliative treatment.

Numerous non-invasive preoperative methods for detection of liver metastases have been proposed and described. Among others computed tomography (CT), magnetic resonance imaging (MRI), Fludeoxyglucose positron emission tomography (FDG-PET), and contrast enhanced ultrasonography. Furthermore, several factors should be considered when choosing a modality for preoperative liver assessment: reproducibility, time consumption, cost, operator dependency, and availability for re-assessment. In Denmark, standard preoperative evaluation in patients with rectal cancer is a CT thorax/abdomen, combined with MRI of the rectum and colonic evaluation by colonoscopy (Sundhedsstyrelsen). In an earlier published study, we reported colonic evaluation in patients with rectal cancer using either magnetic resonance (MR) colonography or standard care (Achiam et al., In press). In this patient population the obligatory MRI of the rectum was combined with MR colonography and diffusion weighted MR (DWMR) of the liver on the same day as CT thorax/abdomen.

The aim of this study was to evaluate the feasibility of DWMR imaging of the liver as part of a combined MRI evaluation of patients with rectal cancers and compare it to the standard preoperative evaluation of the liver with CT.

## Materials and Methods

### Study population

From October 2010 to February 2013, consecutive patients diagnosed with rectal cancers were asked to participate in the study. The current study is a secondary analysis with different endpoints than the primary study, design and eligibility criteria are described earlier (Achiam et al., In press). The patients were randomized to either Group-1: standard preoperative diagnostic liver evaluation (CT of thorax/abdomen) or Group-2: preoperative MR diagnostic work-up (CT of thorax/abdomen and DWMR of the liver). CT and DWMR were carried out on the same day. Randomization was performed using the website www.random.org. A computer-generated randomization schedule using fixed size blocks assigned patients to either group-1 or group-2 using a 1:1 ratio.

The primary endpoint of this study was the feasibility of DWMR imaging of the liver as part of a combined MRI evaluation of patients with rectal cancers. The secondary endpoints were a per-patient and per-lesion analysis for CT and DWMR imaging of liver metastases and a comparison of the two modalities.

### Evaluation

Preoperative CT and DWMR were compared to contrast enhanced laparoscopic ultrasound (CELUS), open liver surgery with ultrasound or consensus reached at the multidisciplinary team conference if surgery was not performed within six months. Contrast enhanced ultrasound was chosen as the control measure to compare for both of CT scan and MRI, since it could be performed as an intraoperative procedure in both groups. Furthermore, studies have found additional metastases and high sensitivities in evaluating especially smaller liver metastases with intraoperative contrast enhanced ultrasound (D’Onofrio et al., 2009; Wildi et al., 2008). The multidisciplinary team board consisted of highly specialized consultants from the specialties: colorectal surgery, hepatobiliary surgery, radiology, oncology and pathology. Furthermore, the patients were monitored at a postoperative follow-up visit. The postoperative follow-up consisted of a visit at an out-patient unit and further diagnostics (CT, contrast enhanced ultrasonography or FDG-PET/CT) if the patient had symptoms of recurrence or a carcinoembryonic antigen (CEA) value >4 mg/L. To be considered true positive finding, a lesion had to be within the same or adjacent liver segment and had to have the same size ±25% as a lesion found on CELUS or supplemental scans. All metastases were defined from a histopatological confirmation when possible or based on the morphological characteristics according to diagnostic modality if no surgical specimen was available. Liver metastases were defined as synchronous metastases, if they were either confirmed or found on CELUS, supplemental scans or subsequent follow-up scans within six months. Liver metastases found after six months on follow-up were defined as metachronous metastases.

### CT technique

The CT examination of thorax, abdomen and pelvis were performed on Philips Brilliance Premium 64 slice, Philips Brilliance 64 slice, or Philips MX 8000 IDT (16 slice) machines (Brilliance; Philips Healthcare, DA Best, the Netherlands). Intravenous contrast, Iomeprol 350 mgI/ml, according to bodyweight, was injected prior to the examination. The examination was performed at 60–70 s timed delay to obtain the portovenous contrast phase. The department’s usual technical parameters were used for the examination; 120 kV and 300 mAs, with 64 × 0.625 mm or 16 × 1.5 mm collimation, reconstructed to 3 mm in axial, coronal and sagittal plane. Hypodense lesions in the parenchyma which showed no sign of being a cyst or haemangioma in the portal-venous phase of a contrast enhanced CT were considered liver metastases. Finding of metastatic lesions were documented and recorded by the size and segmental location in the liver.

### MRI technique

All MRI examinations were performed on a 1.5T system (Achieva, Philips Healthcare, Best, the Netherlands) in three stations: pelvic, liver and bowels. For liver, a 16 channel phased array coil was used and an axial T2-weighted respiratory triggered Single Shot Turbo Spin Echo sequence (time to echo (TE) 80 ms, field of view (FOV) 430 × 330 mm, matrix 300 × 206, 7 mm slice thickness acquisitions time (TA) 1:15 min), a breath hold axial dual echo gradient echo (GE) sequence, time to repeat (TR)/TE/TE: 142/2.3/4.6 ms, FOV 400 × 327 mm, matrix 308 × 193, 5 mm slice thickness, TA 38 s; a respiratory triggered axial DWI (TR/TE 1301/56 ms FOV, 400 × 322 mm, matrix 144 × 94, *b*-values of 0, 10, 150, 300, 450, 8 mm slice thickness, TA 3 min. Apparent Diffusion Coefficient map was calculated on basis of b 0, 150, 300 and 450. Furthermore, an axial breath hold T1w fat saturated GE sequence was obtained before and after intravenous injection of 0.1 mmol/kg body weight gadoterate meglumin TR/TE 4/1.95 ms, FOV 375 × 295 mm, matrix 188 × 147, 4 mm slice thickness TA 20.3 s, followed by a coronal breath hold T1w GE fat saturated sequence TR/TE 4/1.95 ms, FOV 375 × 375 mm, matrix 192 × 192, 4 mm slice thickness, TA 22 s. Hypointense lesions on T1w, variable hyperintense on T2w, hyperintense on DWI and hypointense on ADC map were considered liver metastases. Finding of metastatic lesions were documented and recorded by the size and segmental location in the liver.

### Contrast enhanced laparoscopic ultrasound

Laparoscopic ultrasound was performed via one of the 10-mm laparoscopic ports using a dedicated ultrasound probe with a flexible tip that allows good contact between the liver surface and the probe tip with the curved array transducer (Transducer type 8666; BK Medical Ultrasound System, Herlev, Denmark). The entire liver was thorough examined for focal lesions with conventional B-mode and with contrast mode. Approximately 2.4 ml of ultrasound contrast agent (Sonovue, Bracco, Milan, Italy) was given intravenously and laparoscopic ultrasound (with the system in Low-MI contrast-mode) was performed after 30 s (in the venous phase). Solid liver lesions showing hypovascularity (wash out) were considered liver metastases. Finding of metastatic lesions were documented and recorded by the size and segmental location in the liver.

### Image analysis

CT-images were, as part of the standard operating procedure for patients with rectal cancer, evaluated by radiologists assigned to the abdominal team. The radiologist, who was on duty the day the CT-scan was performed, evaluated the standard CT of the liver.

MR images of the liver were anonymized and randomized by a computer before being blindly evaluated by two evaluators. One reader was a board-certified abdominal radiologist with more than 15 years of experience in MR abdominal imaging, but with limited experience interpreting DWMR. The other reader was an experienced research radiographer with more than 10 years of experience in abdominal imaging and extensive experience evaluating and designing DWMR scan sequences.

CELUS were evaluated by three board-certified abdominal radiologists specialized in ultrasonography examination, each with more than 15 years of experience in abdominal ultrasonography.

## Statistical Analysis

For categorical and continuous data, Fisher’s exact test, Pearson *X*^2^ test and Mann–Whitney *U* test were used when appropriate. SPSS version 19 (IBM version 20.0) was used for the statistical analyses. *P*-values <0.05 were regarded significant.

## Ethics

The study was approved by the research ethics committee, Capital Region of Denmark (protocol number: H-1-2009-094) and registered at clinicaltrials.gov (NCT01544452) and all patients participated after informed consent.

## Results

**Figure 1:**
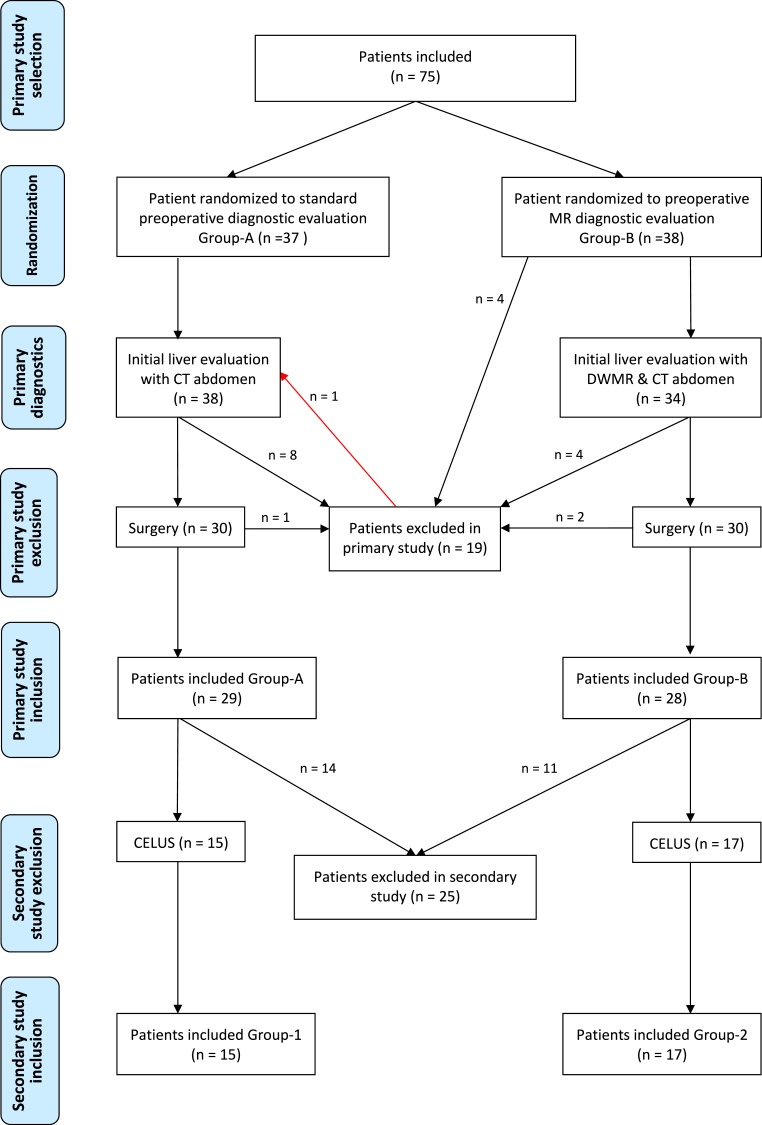
Flowchart of patients included. DWMR, Diffusion weighted magnetic resonance; CELUS, Contrast enhanced laparoscopic ultrasound.

Initially, 75 patients were included in the primary study of which 19 were excluded (reasons are as follows: Patients excluded due to: 6 not undergoing operation, 3 due to benign tumor, 3 due to liver metastases, 2 withdrawal of consent, 1 due to MR missing capacity, 1 due to sigmoid cancer, 1 due to preoperative death, 1 due to CT technical difficulties, 1 due to MR technical difficulties) (Achiam et al., In press). In total 56 patients were included in the primary study. One patient, randomized to Group-2, was excluded in the primary study, since the MR scanner had technical difficulties after the MR sequence of the rectum. Thus, the MR colonography and DWMR were not performed. However, since the patient both had a standard CT thorax/abdomen and a CELUS, the patient was allocated to Group-1 in the current study (Fig. 1).

From the 57 patients, 22 patients were excluded as they did not have surgery and a CELUS or open ultrasound within six months or a multidisciplinary team consensus. Thus, Group-1 consisted of 15 patients having the standard preoperative evaluation of the liver, and Group-2 consisted of 20 (16 male, 4 female) patients having the standard CT evaluation of the liver and DWMR of the liver. Age, sex, and T- and N-stage were comparable between the groups and are shown in Table 1.

**Table 1:** Patient characteristic in Group-1 and 2.

		Group-1		Group-2		*P*-value
		*N*	%	*N*	%	
Sex						0.266^a^
	Female	6	40.0	4	20.0	
	Male	9	60.0	16	80.0	
Age	Median (IQR)	62 (19)		64 (17)		0.657^b^
Preoperative T-stage						1.000^a^
	2	5	33.3	7	35.0	
	3	10	66.7	13	65.0	
Preoperative N-stage						1.000^a^
	0	8	53.3	11	55.0	
	1 & 2	7	46.7	9	45.0	

**Notes.**

IQR Interquartile range

a Fisher’s exact test.

b Mann–Whitney *U* test.

### Group-1

#### CT per-patient analysis

Compared to CELUS findings, CT of the liver correctly identified one patient with liver metastasis. Another patient had liver metastases, all of which were not revealed. No patients had false positive findings (Table 2). The sensitivity, specificity, positive predictive value (PPV) and negative predictive value (NPV) are shown in Table 3.

**Table 2:** Findings on CT and diffusion weighted MR.


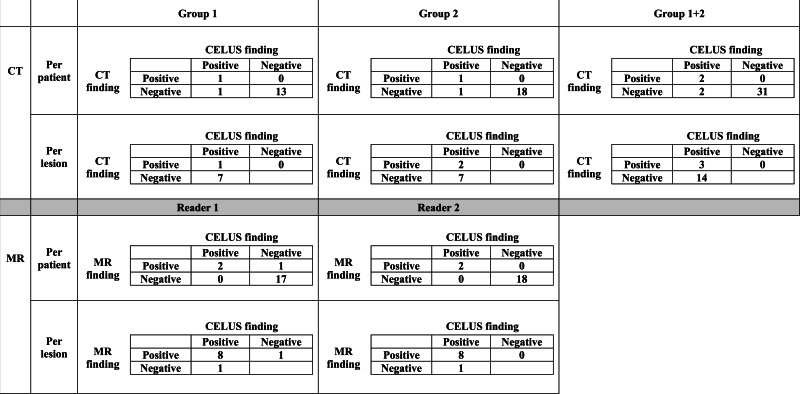

**Notes.**

CELUS Contrast enhance laparoscopic ultrasound

**Table 3:** Per-patient and per-lesion analysis for CT and diffusion weighted MR.

	Per-patient analysis					Per-lesion analysis				
	CT Group 1	CT Group 2	CT Group 1+2	MR Reader 1	MR Reader 2	CT Group 1	CT Group 2	CT Group 1+2	MR Reader 1	MR Reader 2
Sensitivity	50%	50%	50%	100%	100%	13%	22%	17%	89%	89%
Specificity	100%	100%	100%	94%	100%					
PPV	100%	100%	100%	67%	100%	100%	100%	100%	89%	100%
NPV	93%	95%	94%	100%	100%					

**Notes.**

Sensitivity True positive/true positive + false negative Specificity True negative/true negative + false positive Positive predictive value (PPV) True positive/true positive + false positive Negative predictive value (NPV) True negative/true negative + false negative

#### CT per-lesion analysis

Compared to CELUS findings, one of 8 synchronous liver metastases was correctly identified and no false positive lesions were recorded on CT (Table 2). The sensitivity and PPV are shown in Table 3. The lesions are described in Table 4.

**Table 4:** Characteristics of synchronous liver metastases.

Patient number	Group	Lesion no	CT segment/size	DWMR Reader 1 Segment/size	DWMR Reader 2 Segment/size	IOCEUS/MDT Segment/size
1	1	1	NF	–	–	Segment 5/6 mm
		2	Segment 7/12 mm	–	–	Segment 7/20 mm
2	1	1	NF	–	–	Segment 3/8 mm
		2	NF	–	–	Segment 6/6 mm
		3	NF	–	–	Segment 6/7 mm
		4	NF	–	–	Segment 7/10 mm
		5	NF	–	–	Segment 8/5 mm
		6	NF	–	–	Segment 8/8 mm
3	2	1	Segment 4/55 mm	Segment 4/65 mm	Segment 4/70 mm	Segment 4/68 mm
		2	NF	Segment 5/8 mm	Segment 5/5 mm	Segment 5/6 mm
		3	NF	Segment 5/7 mm	Segment 5/7 mm	Segment 5/7 mm
		4	Segment 7/13 mm	Segment 7/18 mm	Segment 7/10 mm	Segment 7/13 mm
		5	NF	Segment 8/11 mm	Segment 8/7 mm	Segment 8/7 mm
		6	NF	Segment 8/11 mm	Segment 8/12 mm	Segment 8/11 mm
		7	NF	Segment 8/13 mm	Segment 8/16 mm	Segment 8/19 mm
4	2	1	NF	Segment 5/23 mm	Segment 5/25 mm	Segment 5/17 mm
		3	NF	NF	NF	Segment 5/14 mm

**Notes.**

DWMR Diffusion weighted magnetic resonance MDT Multidisciplinary team NF not found

#### Consequence of CT findings

One patient had a true positive metastasis of 12 mm in segment-7, but during CELUS a 6 mm metastasis in segment-5 was also identified. Surgical strategy was changed, both metastases were treated with RFA and at the 12 months follow-up no sign of recurrence was found.

Another patient was found without metastases on CT of the liver. However, at the CELUS, several metastases (8 mm segment-3, 6 & 7 mm segment-6, 10 mm segment-7, 8 & 5 mm segment-8) were found. The patient was committed to transarterial chemoembolization FOLFOX-TACE treatment (Martin et al., 2009) and after five months the patient no longer had identifiable metastases.

#### Time from scan to operation

The median time from CT-scan to operation was 2 weeks (range 1–20).

### Group-2

#### CT per-patient analysis

Compared to CELUS findings, CT of the liver correctly identified one patient with liver metastasis. One patient had liver metastases which were not seen on CT. No patients with false positive findings were found (Table 2). The sensitivity, specificity, PPV and NPV are shown in Table 3.

#### CT per-lesion analysis

Compared to CELUS findings, two of nine synchronous liver metastases were correctly identified and no false positive lesions were recorded on CT (Table 2). The sensitivity, specificity, PPV and NPV are shown in Table 3. The lesions are described in Table 4.

#### MR per-patient analysis

Compared to CELUS findings, Reader 1 identified the two patients with liver metastases. No patients with liver metastases were missed. One patient had a false positive finding (Table 2). Reader 2 identified the two patients with liver metastases. No patients with liver metastases were missed and/or had false positive findings. The sensitivity, specificity, PPV and NPV are shown in Table 3.

#### MR per-lesion analysis

**Figure 2:**
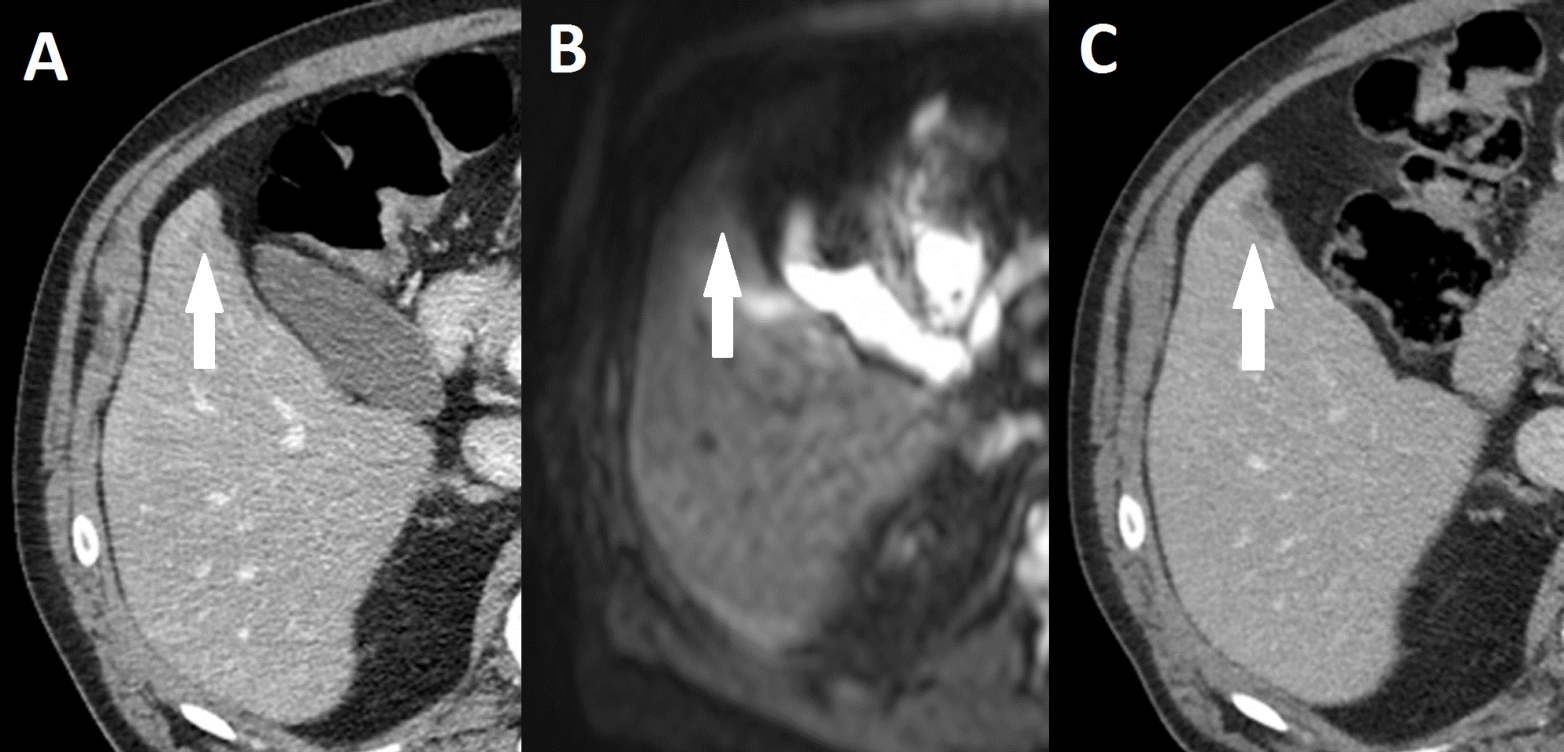
Missed liver metastasis on CT and diffusion weighted MR. (A) Initial CT, (B) Diffusion weighted MR, (C) subsequent CT after 5 months of neoadjuvant therapy. Broken arrow: Metastasis missed on primary CT and diffusion weighted MR.

Compared to CELUS findings, Reader 1 correctly identified eight liver metastases. One lesion was overlooked (Fig. 2) and one false positive lesion was reported (Table 2). Reader 2 also identified eight out of eight liver metastases. A single lesion was overlooked and no false positive lesions were reported. The sensitivity and PPV are shown in Table 3.

#### Consequences of DWMR findings

**Figure 3:**
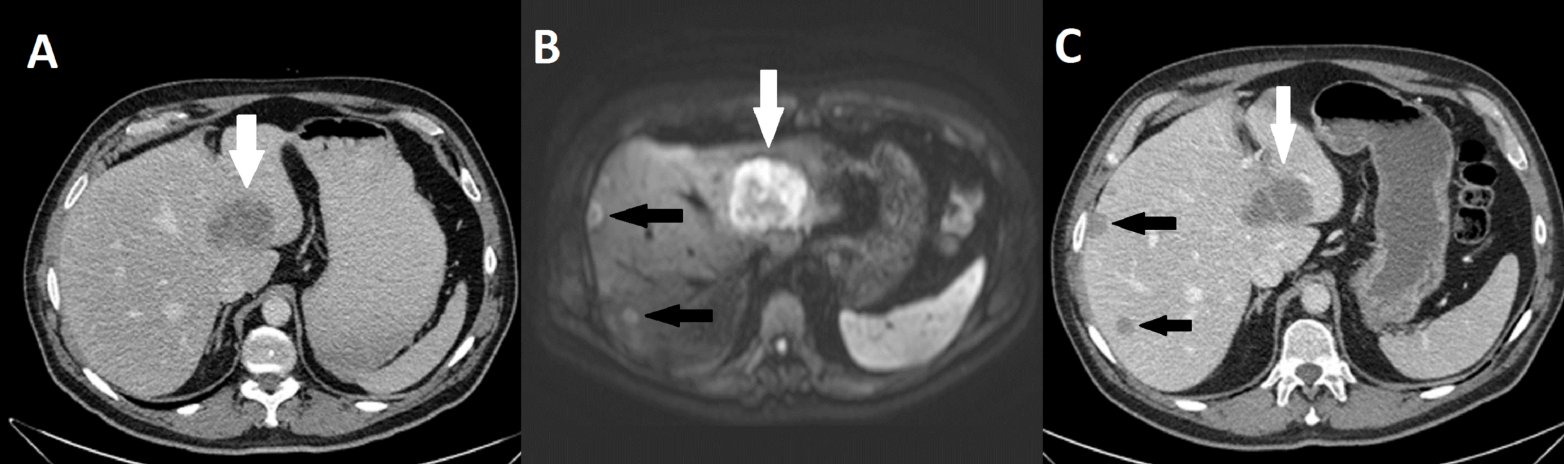
Missed liver metastasis segment 7 & 8 on CT and diffusion weighted MR. (A) Initial CT, (B) Diffusion weighted MR, (C) CT in venous phase. White arrow: Segment 4 metastasis. Black arrow: Two metastases missed on primary CT.

In one patient with rectal cancer, two liver metastases (55 mm segment-4 and 13 mm segment-8), were initially found to be resectable (Fig. 3) on CT of the liver. However, DWMR subsequently found multiple metastases (Table 3 and Fig. 3). On the basis of the finding on DWMR, the metastases were found to be non-resectable and surgery was cancelled. Instead, the patient was committed to neoadjuvant treatment using transarterial chemoembolization with FOLFOX. After four months of treatment, the patient underwent a liver resection with findings of metastases in segment 4, 5, 6, and 8. The patient had a rectum extirpation two months later and at the 12 months follow-up, no sign of recurrence was found.

**Figure 4:**
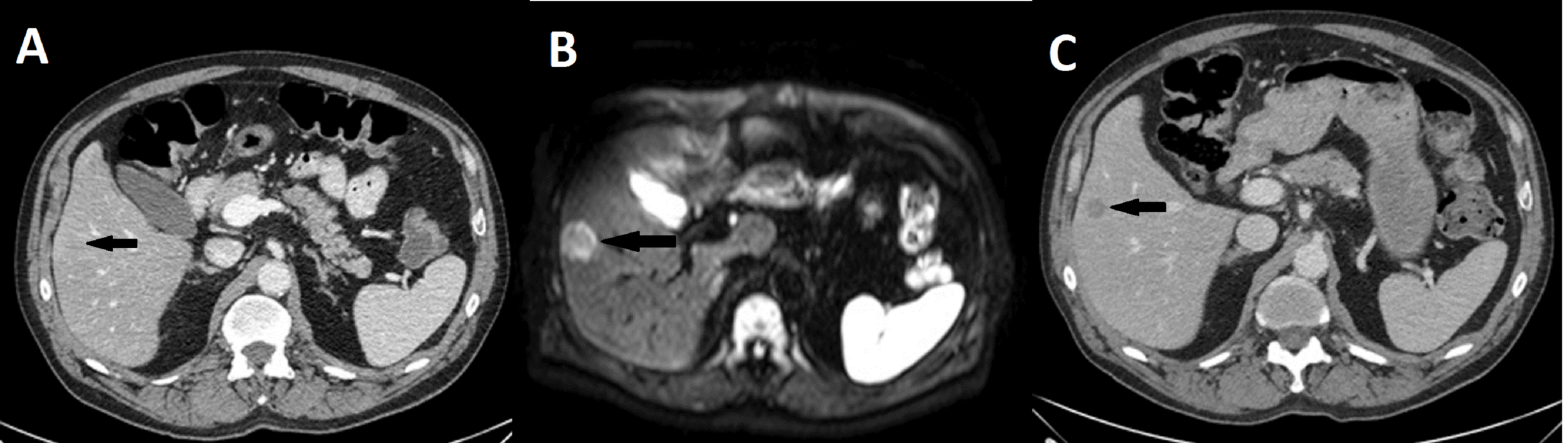
Missed metastasis on initial CT and diffusion weighted MR. (A) Initial CT, (B) Diffusion weighted MR, (C) CT in venous phase after 3 months of neoadjuvant therapy. Black arrow: Metastasis missed on primary CT.

In another patient with rectal cancer, no liver metastases were found on the initial CT scan, but retroperitoneal metastases were found. Subsequently, a liver metastasis (23 mm segment-5) was found on the DWMR (Fig. 4). On the basis of the finding on DWMR and CT the patient was committed to neoadjuvant treatment and underwent rectum resection after seven months of treatment. Eight months after surgery the patient was diagnosed with multiple metastases in the lungs, but no liver metastases. At the last follow-up scan (26 months postoperatively) no liver metastases were detected.

One patient had a false positive finding according to Reader 1, however no lesion was found on the subsequent CELUS and no change of strategy was implemented.

#### Time from scan to operation

The median time from CT and DWMR to surgery was 2.5 weeks (range 1–33).

### CT versus DWMR

The sensitivity, specificity, PPV and NPV of the combined CT findings from Groups-1 and -2 are shown in Table 3. Comparing lesions confirmed on CELUS (9), significantly more true positive lesions diagnosed found on DWMR (8) compared with lesions found on CT (1) on per-lesion analysis (*p* = 0.001 Reader 1, *p* = 0.003 Reader 2, Fisher’s exact test). However, on per-patient analysis compared to confirmed lesions on CELUS (20), no difference was found (*p* = 0.429, Fisher’s exact test) between the lesions found on DWMR (20) and the lesions found on CT (14 of 15).

### Follow-up scans

The median time from surgery until the last follow-up scan was 25 months (range 2–48) and 23 (range 1–46) in Groups-1 and -2, respectively. The type of control scan is shown in Table 5.

**Table 5:** Type of follow-up scan.

Group	Follow-up scan	Frequency	Percentage
	CT	7	47
1	PET/CT	2	13
	Contrast enhanced ultrasound	6	40
	Total	15	100
2	CT	9	45
	None due to comorbidity	1	5
	PET/CT	2	10
	Contrast enhanced ultrasound	8	40
	Total	20	100

In Group-1, follow-up scan compared to preoperative CT and CELUS revealed a patient with additional synchronous liver metastases found two months after surgery. Furthermore, one 38 mm metachronous liver metastasis was found after 21 months in another patient in Group-1.

Two patients in Group-2 were found with metachronous metastases on follow-up scans. One patient with multiple metachronous liver lesions (5, 5, 18, 22 mm) was found after 15 months and one patient with one metachronous metastasis (30 mm) after 14 months. One patient did not undergo control scans due to severe co-morbidity.

## Discussion

The primary finding of this study was a per-patient sensitivity/specificity of 50/100% for CT. For DWMR, the per-patient sensitivity/specificity was 100/94% and 100/100% for Reader 1 and 2, respectively. The per-lesion sensitivity of CT and DWMR were 17% and 89%, respectively. Furthermore, one patient had non-resectable metastases after DWMR, despite being diagnosed with resectable metastases after CT. Another patient was diagnosed with multiple liver metastases during CELUS, despite a negative CT-scan.

The results of our study are in line with recent large meta-analyses which recommended MRI for diagnosis of colorectal liver metastases (Floriani et al., 2010; Niekel, Bipat & Stoker, 2010). In the latest meta-analysis comparing the modalities of CT, MRI and FDG-PET, a pooled per-patient sensitivity and specificity of 75% and 96% for CT compared to 81% and 97% for MRI, and 94% and 99% for FDG-PET were found. The per-lesion sensitivity was 83% for CT, 86% for MRI and 86% for FDG-PET (Floriani et al., 2010). However, both in the per-patient and per-lesion analyses of the diagnostic modalities of MRI vs CT, the odds ratio [OR] (95% confidence interval) were in favor of MR with an OR of 0.69 (0.47–0.99) and 0.66 (0.55–0.80), respectively. Furthermore, using liver specific contrast agents in the per-lesions analysis, the difference was even more pronounced (Floriani et al., 2010). To our knowledge no studies have been published comparing CT and DWMR of the liver for diagnosis of liver metastases for colorectal cancer, but several trials have shown higher sensitivities of DWMR compared to conventional MR imaging or MR with liver specific contrast (Parikh et al., 2008; Nasu et al., 2006).

The importance of preoperative evaluation of the liver cannot be overstated since surgical treatment has been shown to have the best long-term survival rates. One study found disease free survival at 5 and 10 years of 27% and 22%, respectively, with an overall survival at 5 and 10 years being 47% and 28%, respectively (Wei et al., 2006). Furthermore, there are other treatments, including RFA and perioperative chemotherapy combined with surgery, which have a 4-year overall survival of 20–49% (Lee et al., 2008; Park et al., 2008) and a 5-year overall survival of 51% (Nordlinger et al., 2013), respectively. This is to be compared with patients with colorectal cancers having a 5-year overall survival of 3.3% for untreated synchronous liver metastases (Manfredi et al., 2006) or a mean survival of 8.5 months (Gorog, Toth & Weltner, 1997). However, common for all treatment strategies and prognoses is that it solely relies on accurate staging, especially for diagnosis of liver metastases.

The results of this trial also show that the sensitivity of contrast enhanced ultrasound performed during surgery, as shown in several other studies (Shah et al., 2010; Zacherl et al., 2002), was high and will find new liver metastases compared to preoperative staging. Two trials reported at least one additional malignant lesion found on intraoperative ultrasound in 12–33% of cases where pre-contrast CT followed by post-contrast CT scans were used (Shah et al., 2010; Zacherl et al., 2002). However, an even more important finding of the studies, also underlined in this study, was that liver metastases could and should be diagnosed prior to surgery as they may alter the treatment strategy. In the two studies, change of surgical strategy was seen in 14–23% of the cases due to intraoperative ultrasound findings (Shah et al., 2010; Zacherl et al., 2002). In this study, 3/15 (20%) patients were inadequately diagnosed preoperatively with CT. One patient was found inoperable after DWMR and thus underwent new adjuvant therapy before liver resection, one patient had additional RFA and in the last case the patient underwent postoperative chemotherapy as the lesions were not detected preoperatively. In worst case scenarios inadequate preoperative assessment of the liver may result in futile laparotomies. This has been described in up to 11% of patients with R0 colorectal cancer resections and liver metastases who had a preoperative evaluation with CT (Ruers et al., 2009). The fact, that CT of the liver may not be an adequate preoperative evaluation was demonstrated in the same study, which also found a 60% potential reduction in futile laparotomies by adding FDG-PET to CT-scan in the preoperative evaluation (Ruers et al., 2009). Besides a futile laparotomy, more serious consequences arise from inadequate preoperative staging as patients undergoing surgery with undetected liver metastases may progress from having resectable metastases to non-resectable metastases. One obvious cause of progression is the prevention or the delay in chemotherapy due to postoperative complications. Complications may occur after any operation and especially in rectal resections, which has been shown to have anastomotic insufficiency in up to 13% (DCCG). Another hypothetical cause of progression in the liver metastases may be the surgical stress following laparotomy. The degree of surgical stress is proportional to the postoperative immunological decrease in the body (Baigrie et al., 1992). These immunological changes lead to impaired immune function which may lead to increased postoperative complications and increased morbidity (Baigrie et al., 1992; Ohzato et al., 1992; Hensler et al., 1998). Furthermore, it has been shown, that a decrease in cell mediated immune function by cytotoxic T-lymphocytes and NK-cells contribute to an increased susceptibility to the growth of metastases in oncological patients having rectal cancer (Koda et al., 2003; Kondo et al., 2003).

The weakness of the study is that only 57 patients were included in a period of three years. Despite all efforts of recruiting patients consecutively, logistic problems resulted in a low accrual rate. This may be a potential bias, but our patients were prospectively and blinded randomized to minimize bias. Another weakness of the study was the fact, that only 33 out of the 57 patients had CELUS and that assessment of potential missed liver metastases were dependent on postoperative follow-up with a CT-scan. The results may therefore be an overestimation of the sensitivity. The different modality used for the postoperative control is another weakness which may yet again lead to an overestimation of sensitivity and specificity. Furthermore, the fact that the radiologist who evaluated the CT images was the one who was on duty, may have caused a bias in the outcome, especially since the readers of DWMR and CELUS were dedicated and experienced readers.

## Conclusions

Our study found DWMR to be feasible as a preoperative evaluation of liver metastases. Furthermore, our study has shown potential disadvantages as a result of the current standard preoperative evaluation by CT-scan. We recommend that patients with rectal cancer, who are scheduled for MR of the rectum, should have a DWMR of the liver performed at the same time.

## Supplemental Information

10.7717/peerj.1532/supp-1 Supplemental Information 1 Prospective study ofpreoperative full MRI evaluation vs. standard preoperative evaluation inpatient with rectal cancer. Original trial protocol

10.7717/peerj.1532/supp-2 Supplemental Information 2 Dataset.

10.7717/peerj.1532/supp-3 Supplemental Information 3 CONSORT check list.

## Abbreviations

CEA: Carcinoembryonic antigen
CELUS: Contrast enhanced laparoscopic ultrasound
CT: Computed tomography
DWMR: Diffusion weighted magnetic resonance
FDG-PET: Fludeoxyglucose positron emission tomography
FOV: Field of view
GE: Gradient echo
MR: Magnetic resonance
NPV: Negative predictive value
PPV: Positive predictive value
RFA: Radiofrequency ablation
TA: Acquisitions time
TE: Time to echo
TR: Time to repeat

## Additional Information and Declarations

### Data Availability

The following information was supplied regarding data availability: The research in this article did not generate any raw data.

### Clinical Trial Registration

The following information was supplied regarding Clinical Trial registration: Clinicaltrials.gov (NCT01544452).
