# Supplementary material for: Preoperative CT versus diffusion weighted magnetic resonance imaging of the liver in patients with rectal cancer; a prospective randomized trial
Source: PeerJ. 2016 Jan 14;4:e1532. doi: 10.7717/peerj.1532 (PMC4715462; doi:10.7717/peerj.1532)
Supplement: Supplemental Information 1 — Original trial protocol [file peerj-04-1532-s001.doc]

**Prospective study of preoperative full MRI evaluation vs. standard preoperative evaluation in patient with rectal cancer**

**Study goals and objective**

To investigate the quality of preoperative evaluation in patients with rectal cancer, either as a full MR imaging (MRI) with MR rectum + MR colonography + MR liver and chest CT versus current standard preoperative evaluation (MR  rectum + endoscopy + abdominal /chest CT). Furthermore, to assess the economic aspects of full preoperative MRI evaluation vs. standard preoperative evaluation in patients with rectal cancer.

**Background**

Colorectal cancer (CRC) is among the most common cancer forms in Denmark with an approximate of 4300 new cases in Denmark annually, of which rectal cancer represents approximately 1400 new cases each year [1]. It is also well known that synchronous cancer and polyps are present in up to 11% and 58% in patients with CRC [2, 3, 4, 5, 6bv]. It is assumed that adenomas constitute a precursor for cancer [7] and it is thus speculated that the detection and removal of adenomas could reduce the incidence and mortality of colorectal cancer. Danish Colorectal Cancer Group (DCCG) and the Danish Surgical Society (DKS) currently recommend full colonic investigation as part of the preoperative evaluation, which also includes MRI of the rectum, ultrasound of the liver and chest X-ray (or abdominal /chest CT) to locate possible metastasis or synchronous tumors [8]. However, it is often difficult to implement the preoperative colonic investigation due to lack of capacity or tumor stenosis. A recent Danish study showed that up to 78% of all patients with colorectal cancer had not received the full colonic investigation preoperatively [9]. In this instance, the recommendation from DCCG & DKS is that patients, in the absence of complete colonic investigation preoperatively, should undergo colonoscopy within three months postoperatively.

Within the last 15 years new non-invasive imaging techniques have been developed, this includes MR colonography (MRC). Like conventional colonoscopy, ​​MRC requires bowel cleansing, since feces can create artifacts that can hide or mimic polyps and abnormalities. After colonic cleansing the colon is distended by water using a rectal catheter. Since it is only water that needs to pass through a possibly stenotic colon segment, there is a better chance to successfully examine the entire colon compared to a colonoscopy. A recent study showed a 98% success rate using MRC to examine the entire colon in patients with CRC having colon stenosis [10]. The MRC is preformed after the colon is fully distended with water and the scan times are between 10 and 15 minutes. Data processing, reconstruction and analysis are made at an independent workstation.

The advantages of MRC are its non-invasive nature, short examination time, and the fact that sedation is unnecessary. This makes it possible for patients to be discharged directly after the imaging procedure as opposed to the necessity for admission after a colonoscopy until the effects of the sedative drugs have worn off. Furthermore, it is assumed that patient compliance is much higher in MRC, since the majority of the patients find it less unpleasant than colonoscopy [11, 12].
MRI of the liver is a well-known procedure that has shown good results in the diagnosis of hepatic metastases and primary cancers. Several studies have shown that it is equal or better than CT and ultrasound of the liver [13, 14].

Currently there are no studies, which make the overall preoperative assessment by means of one investigating technique, namely MRI. We have previously studied the sensitivity/specificity and patient satisfaction by MRC with fecal tagging [15]. In this study we want to investigate the quality of MRC with bowel cleansing, also assessing the economic aspects of an overall examination of the rectum, colon and liver in patients with rectal cancer.

**Design**

Prospective randomized clinical trial. Patients diagnosed with rectal cancer are invited to participate in a full preoperative MRI investigation (rectum, liver ad colonography) + chest CT or to participate in the standard preoperative evaluation (rectal MRI, +/- endoscopy and abdominal/thoracic CT) to exclude metastasis. The randomization is computerized.

The studies are evaluated by two blinded investigators. All patients will also receive an intraoperative ultrasound of the liver, which will serve as the gold standard for liver examination.

**Study population:**

140 patients with histopathological confirmed rectal cancer, scheduled for surgery.

**Inclusion criteria:**

Patients with rectal cancer scheduled for surgery.

**Exclusion criteria:**

Missing informed consent

IBD (Inflammatory bowel disease)

Pacemaker

Metal parts in the body

Claustrophobia

Psychiatric disorders

Age under 18

Pregnancy

Kidney diseases

Heart disorders (i.e. arrhythmia)

**Methods:**

Patients scheduled for surgery after histology confirmed rectal cancer are invited to participate in the study after obtaining an informed consent. After acceptance patients are randomized by a computer generated randomization code, to either receive the full preoperative MRI evaluation (rectum, liver ad colonography) + chest CT or to participate in the standard preoperative evaluation. The patients are randomized in blocks of groups of 10 patients. Both groups will also receive an intraoperative ultrasound of the liver, which will serve as the gold standard for liver examination.  After the first 140 patients are included in the study, additional participants will be included in blocks of 10 if patients have dropped out of the study. This will ensure that the overall enrolled number of patients who have completed the project will be at a minimum of 140.

The day before the full MRI investigation, patients have to carry out a colonic cleansing, following the guidelines of bowel cleansing with Moviprep®.

The patients not included in the study will receive the standard preoperative evaluation.

During the procedure the patient is placed in supine position, where a rectum scan will be performed followed by a liver scan. Hereafter, the colon will be distended with water by a rectal catheter and the remaining scans will be conducted. Each of the MRC scan sequences are about 20 sec. with patient breath hold during each sequence. MRI contrast agent is administered to emphasize pathology in the T1 MR imaging.

Furthermore, patient will be administered intravenously Buscopan®, to reduce bowel movement and thus imaging artifacts.

Data processing, reconstruction and analysis are made at an independent workstation.

**Safety Considerations:**

Department guidelines are followed. Monitoring for MRI scanning with contrast is performed by radiology technicians and there will be an immediate access to a radiologist in necessary cases.

**Patient recruitment:**

Patients will be recruited in *Gastroenheden*, surgical gastroenterology section, Herlev University Hospital, as a part of their first outpatient visit after having the cancer diagnosis confirmed. The patient will be contacted directly by research year student Talie HadiKhadem or another member of the research group.

**Informed consent:**

All potential study participants will receive oral and written information about the study. Inclusion takes place after obtaining informed consent.

**Risks and side effects:**

The MRI contrast agent (Gadolinium) is used in daily routine MRI scans and is administered intravenously. Side effects are extremely rare and may present as warmth, a metallic taste in the mouth, palpitations, headaches and dizziness associated with the injection and in rare cases hives and nausea.

Buscopan® is also a common agent used in clinical routine practice such as endoscopy with rare side effects. Known side effects are dry mouth, pupil dilation, mucosal dryness, heart palpitations and urinary retention.

Moviprep® is a routine bowel cleansing agent with extremely rare side effects. These includes bloating, nausea; rectal irritation; stomach cramps or pain, vomiting, muscle pain, shivering, diarrhea and dizziness.

There is a possibility of scanner induced loud noises during MRI scanning. Patients will be offered a hearing protection device.

There is always a radiologist in the immediate vicinity during the MRI scans. The scans will take place at the Radiology department at Herlev University Hospital.

**Ethical aspects:**

A full preoperative MRI evaluation has potentially enormous consequences for investigation and diagnosis in patients with colorectal cancer. There are many expected advantages compared to the existing diagnostic tools (as mentioned above) in this procedure if validated and developed.

This applies both to the patient but also on a socioeconomic perspective. During the study, project subjects will receive a more extensive diagnostic investigation.

The expected beneficial effects of the project, both for the individual patient and for future patients, thereby justify the studies predictable risks, side effects and disadvantages.

**Statistics:**

Sample size is calculated to be 50 patients in a group to ensure at least 1 synchronous cancer in each group with a 95% likelihood, assuming that the prevalence of synchronous cancer is 6%. To further strengthen the study the number is increased to 75 patients in each group.

**Logistical framework:**

The project will be performed at the Department of Radiology and Gastroenheden, Herlev University Hospital.

**Approval:**

The project has been approved by the Regional Ethics Committee and the Danish Data Protection Agency.

**Time frame:**

The project is intended to have a time span of 48 month.

**Support:**

The project is thought to be a continuation of part of Ph.D thesis by Michael Achiam "Clinical Aspects of MR Colonography as a Diagnostic Tool" on the initiative of Michael Achiam, Jacob Rosenberg and Henrik Thomsen.

No one in the project group has economic interests in the project and patients will not be accommodated economically.

Reference List

1. Cancerregisteret 2005 og 2006. Nye tal fra Sundhedsstyrelsen, 2008

2. Adloff M, Arnaud JP, Bergamaschi R, Schloegel M. Synchronous carcinoma of the colon and rectum: prognostic and therapeutic implications. Am.J.Surg. 1989; 157 (3):299-302

3. Cunliffe WJ, Hasleton PS, Tweedle DE, Schofield PF. Incidence of synchronous and metachronous colorectal carcinoma. Br.J.Surg. 1984; 71 (12):941-943

4. Isler JT, Brown PC, Lewis FG, Billingham RP. The role of preoperative colonoscopy in colorectal cancer. Dis.Colon Rectum 1987; 30 (6):435-439

5. Langevin JM, Nivatvongs S. The true incidence of synchronous cancer of the large bowel. A prospective study. Am.J.Surg. 1984; 147 (3):330-333

6. Nikoloudis N, Saliangas K, Economou A, Andreadis E, Siminou S, Manna I, Georgakis K, Chrissidis T. Synchronous colorectal cancer. Tech.Coloproctol. 2004; 8 Suppl 1:s177-s179

7. Leslie A, Carey FA, Pratt NR, Steele RJ. The colorectal adenoma-carcinoma sequence. Br.J.Surg. 2002; 89 (7):845-860

8. DCCG og Dansk Kirurgisk Selskab. Diagnostik og behandling af kolorektal cancer, 2005

9. Achiam MP, Burgdorf SK, Wilhelmsen M, Alamili M, Rosenberg J. Inadequate preoperative colonic evaluation for synchronous colorectal cancer. *Scand.J.Surg.* 2008 (In Press)

10. Achiam MP, Logager VB, Chabanova E, Eegholm B, Thomsen HS, Rosenberg J. Diagnostic accuracy of MR colonography with fecal tagging. Abdom.Imaging 2008;

11. Florie J, Birnie E, van Gelder RE, Jensch S, Haberkorn B, Bartelsman JF, van dS, V, Snel P, van dH, V, Bonsel GJ, Bossuyt PM, Stoker J. MR colonography with limited bowel preparation: patient acceptance compared with that of full-preparation colonoscopy. Radiology 2007; 245 (1):150-159

12. Hartmann D, Bassler B, Pfeifer B, Eickhoff A, Weickert U, Riemann JF, Layer G. Patient acceptance of magnetic resonance colonography: a prospective inquiry for comparison to conventional colonoscopy. Dtsch.Med.Wochenschr. 2006; 131 (45):2519-2523

13. Kim YK, Ko SW, Hwang SB, Kim CS, Yu HC. Detection and characterization of liver metastases: 16-slice multidetector computed tomography versus superparamagnetic iron oxide-enhanced magnetic resonance imaging. Eur.Radiol 2006; 16 (6):1337-1345

14. Rappeport ED, Loft A, Berthelsen AK, von der RP, Larsen PN, Mogensen AM, Wettergren A, Rasmussen A, Hillingsoe J, Kirkegaard P, Thomsen C. Contrast-enhanced FDG-PET/CT vs. SPIO-enhanced MRI vs. FDG-PET vs. CT in patients with liver metastases from colorectal cancer: a prospective study with intraoperative confirmation. Acta Radiol 2007; 48 (4):369-378

15. Achiam MP, Logager V, Chabanova E, Thomsen HS, Rosenberg J. Patient acceptance of MR colonography with improved fecal tagging versus conventional colonoscopy. Eur.J.Radiol 2008; [Epub ahead of print]
